# Supplementary material for: An Intron c.103-3T>C Variant of the AMELX Gene Causes Combined Hypomineralized and Hypoplastic Type of Amelogenesis Imperfecta: Case Series and Review of the Literature
Source: Genes (Basel). 2022 Jul 18;13(7):1272. doi: 10.3390/genes13071272 (PMC9321068; doi:10.3390/genes13071272)
Supplement: Supplementary file 1 [file genes-13-01272-s001.zip › genes-1742498-supplementary.pdf]

Supplementary S1:

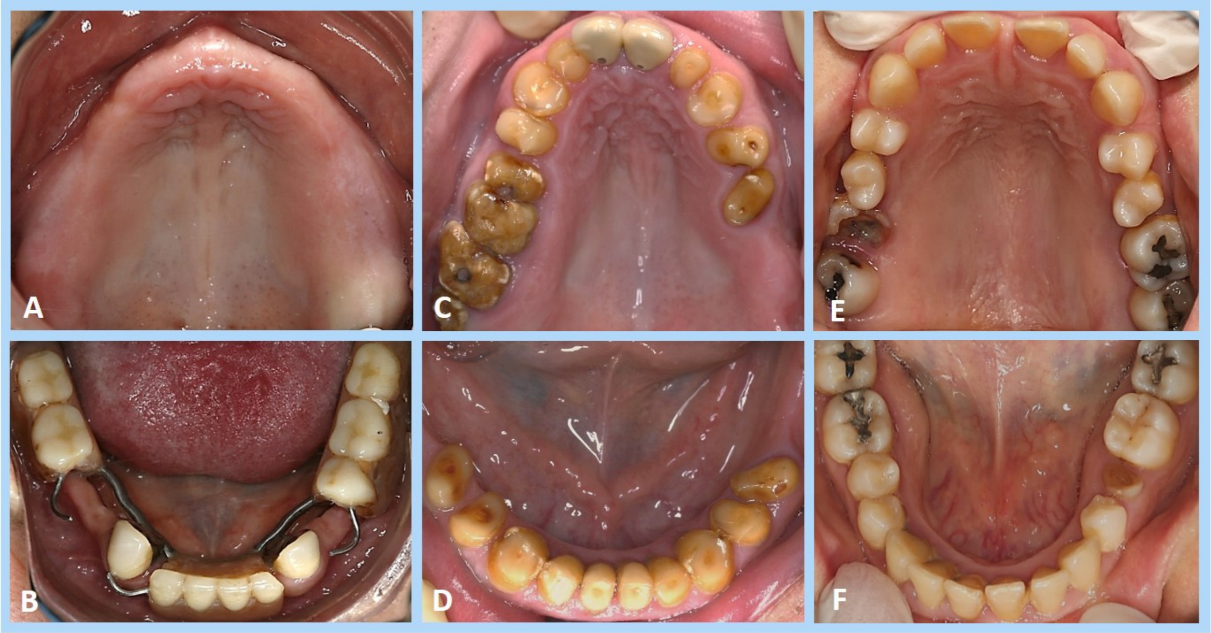

Figure S1. (A) In family I, the proband's mother has no crowns to allow enamel inspection. Her upper jaw is toothless, and (B) only canine teeth and a removable partial denture are present in the lower jaw. A dental examination of other siblings and the father from family I was not possible. In family II, (C-D) the probands' father's teeth show developmentally aberrant enamel, while (E-F) the mother's teeth developed normally.

Supplementary S2: Primers used to amply *AMELX* and *FAM83H* gene

Primers used for the amplification of the relevant parts of the *AMELX* gene were TCCCTGTGTAACCTCAGTCAAG and TCCCATTAATGTCTGCATGTG, and for the *FAM83H* gene GGCCAAGCAGGACTCATTC and GACTCCCCGGAGATGGTAAG. PCR was performed in a volume of 20 ml containing: 1 µl each of forward and reverse primers, 7 µl water, 10 µl Taq Man^{TM} Universal PCR Master Mix Polymerase (PE Applied Biosystems, Piscataway, NJ, USA) and 1 µl DNA. PCR was performed in the GenAmp PCR System 9700 (PE Applied Biosystems, Piscataway, NJ, USA) by an initial denaturation at 94°C for 5 min, followed by 35 cycles of denaturation at 94°C for 30 s, annealing at 58°C (*AMELX*) and 59°C (*FAM83H*) for 30 s, extension at 72°C for 30 s, followed by a final extension at 72°C for 7 min. The PCR products were electrophoresed on 2% agarose gels dyed with 2 mg/ml Sybr Green (30 min, 90 V). After a second electrophoresis of PCR products, the concentration of amplified sequences was estimated. Sequencing PCR was performed in a volume of 20 ml under the following conditions: 25 repeated cycles of denaturation at 96°C for 10 s, annealing at 50°C for 5 s and extension at 60°C for 4 min.

Supplementary S3:

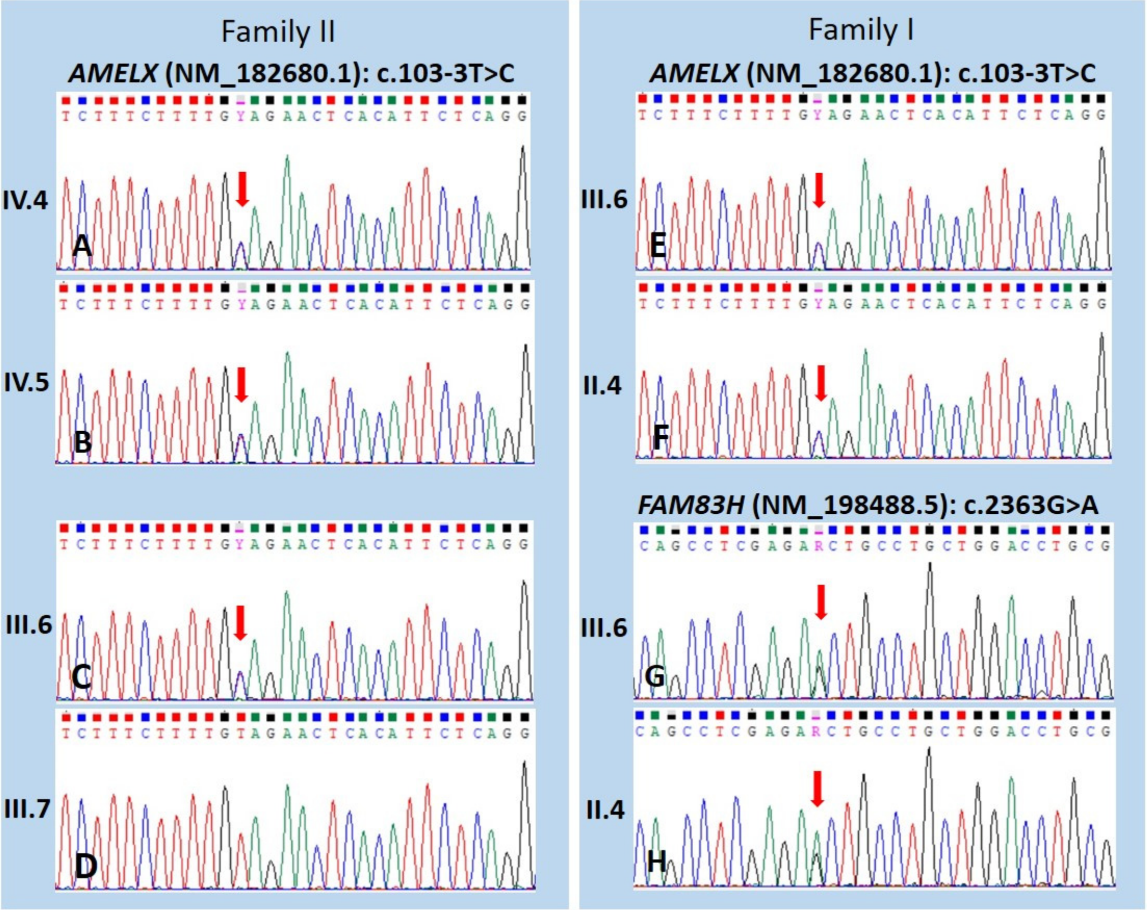

Figure S2. In the family I and family II, segregation analysis confirmed the following variants of *AMELX* and *FAM83H* (highlighted by red arrows). The presence of a disease-causing heterozygous variant of *AMELX* c.103-3T>C was detected in (A) the proband (IV.4), (B) her younger sister (IV.5) and (C) their father (III.6) but not in (D) their mother (III.7) from the family II, and in (E) the proband (III.6) and (F) her mother (II.4) from the family I. Heterozygous *FAM83H* variant c.2363G>A was detected in the family I only; it was present in (G) the proband (III.6) and (H) her mother (II.4).
